# Impact of the prolonged economic crisis on healthcare delivery and workforce resilience in the Kurdistan Region of Iraq: a qualitative study

**DOI:** 10.1186/s12913-026-14147-4

**Published:** 2026-02-05

**Authors:** Kochr Ali Mahmood, Araz Qadir Abdalla, Govand Saadadin Sadraldeen, Dawan Jamal Hawezy, Gulala Ismail M-Amin, Sirwan Khalid Ahmed, Rawand Abdulrahman Esssa, Ardalan Jabbar Abdullah

**Affiliations:** 1 Faculty of General Medicine, Koya University, Koya, Kurdistan region Iraq; 2 College of Nursing, University of Raparin, Rania, Sulaymaniyah, Kurdistan Region 46012 Iraq; 3 Department of Business management, College of Humanity Sciences, University of Raparin, Rania, Sulaymaniyah, Kurdistan Region 46012 Iraq; 4 School of Medicine, University of Kurdistan Hawler, Erbil, Kurdistan region Iraq; 5 Rizgary Teaching Hospital, Erbil, Kurdistan Region Iraq; 6 Department of Nursing, Koya Technical Institute, Erbil Polytechnic University, Erbil, Iraq; 7 Department of Medical Laboratory Science, Faculty of Science, Knowledge University, Erbil, Kurdistan Region Iraq

**Keywords:** Economic crisis, Healthcare delivery, Workforce resilience, Kurdistan Region of Iraq, Salary instability, Resource shortages, Telemedicine, Health system reform

## Abstract

**Background:**

The economic crisis in the Kurdistan Region of Iraq has severely affected public sector salaries and healthcare infrastructure. These disruptions have increased pressures on the healthcare workforce and exposed gaps in the region’s institutional and workforce resilience. This study explored physicians’ experiences and adaptive responses to the prolonged financial instability and its consequences for healthcare delivery.

**Methods:**

A qualitative design was adopted using semi-structured interviews with 25 physicians from various specialties working in both public and private sectors across the Kurdistan Region. The study was conducted from March to August 2024 during a period of heightened financial instability. Data were analyzed thematically using a structured six-step qualitative analysis, using an inductive qualitative thematic analysis approach.

**Results:**

Ten interrelated but analytically distinct themes emerged: workload-related operational stress, salary instability, psychological stress, burnout and hopelessness, coping mechanisms, institutional inaction, clinical decisions under constraints, proposed reforms, need for support, emerging innovations, and impact on patient care. Physicians described increased workloads, delayed or reduced salaries, emotional exhaustion, and a deep sense of hopelessness. Institutional inaction compounded these challenges, forcing doctors to ration care and delay procedures. Despite adversity, participants demonstrated moral resilience through volunteering, free care, and semi-private models. Limited innovations—such as telemedicine and community outreach—were also noted. Collectively, these findings reveal a fragile health system sustained primarily by physicians’ ethical commitment rather than institutional stability.

**Conclusion:**

The Kurdistan healthcare system endures economic hardship through the moral endurance and adaptability of its physicians. Sustaining healthcare quality requires transforming this individual resilience into institutional resilience through salary stabilization, leadership accountability, and structured psychosocial and professional support. Framing these findings through a resilience lens highlights the urgent need to strengthen both workforce resilience and system-level resilience to sustain healthcare delivery during prolonged crises.

## Introduction

Economic crises exert profound and long-lasting effects on health systems through budget contraction, weakened social protection, and reduced access to essential services. Global evidence consistently demonstrates that fiscal instability undermines healthcare infrastructure, increases out-of-pocket spending, and exacerbates health inequities, particularly in systems with limited governance capacity and social safety nets [1–4]. While previous crises, including the 2008 global financial downturn, have revealed how austerity measures can erode workforce stability, reduce service availability, and widen disparities in access to care [5–7], these effects are not uniform across settings. Health systems with strong governance, adaptive financing, and institutional accountability have shown greater capacity to absorb shocks and maintain essential services during periods of economic stress [8].

In contrast to these comparatively well-documented contexts, the Kurdistan Region of Iraq (KRI) represents a distinct and underexplored case of prolonged, recurrent economic crisis within a fragile political and fiscal environment. Since 2014, the region has experienced repeated salary delays, volatile public financing, and constrained investment in health infrastructure driven by oil dependency, budget disputes with the federal government, and political instability [9]. Unlike short-term economic downturns, this sustained fiscal disruption has normalized financial uncertainty within the public sector, fundamentally reshaping employment conditions, service delivery, and professional practice in healthcare. Yet, despite growing evidence on the mental health consequences of economic hardship in the Kurdish population [10], little is known about how such prolonged instability transforms physicians’ clinical decision-making, workforce morale, and institutional adaptation. This study addresses that gap by examining how physicians in the Kurdistan Region of Iraq navigate and respond to chronic economic uncertainty within their daily professional environments.

To conceptually frame these dynamics, the study draws on the notion of *health system resilience*—the capacity of health institutions to absorb shocks, adapt to disruptions, and maintain core functions during crises. Closely related is *organizational resilience*, which emphasizes how leadership, governance, and workplace culture enable frontline staff to cope with and respond to systemic stress. These frameworks provide a useful lens for interpreting how physicians navigate financial instability and how institutional responses—or their absence—shape professional behavior and service delivery. In this study, resilience is operationalized at two interrelated levels: at the individual level, as physicians’ moral endurance, emotional coping, and adaptive professional practices under financial strain; and at the organizational/system level, as the capacity of healthcare institutions and governance structures to absorb economic shocks, support the workforce, and maintain essential services during prolonged crisis Therefore, this study aims to explore how physicians in the Kurdistan Region of Iraq experience, interpret, and adapt to prolonged economic instability, and to examine the implications of these conditions for healthcare delivery and workforce resilience.

## Methods

### Research design and philosophical orientation

This study employed a qualitative exploratory design grounded in a constructivist epistemological perspective, recognizing that experiences are socially situated and co-constructed through interaction between researchers and participants [11]. Data were analyzed using an inductive thematic analysis approach, allowing patterns and meanings to emerge directly from participants’ accounts rather than from predetermined frameworks [11]. This design supported in-depth exploration of physicians’ lived experiences of economic instability and their adaptive responses to systemic constraints. To enhance methodological transparency and rigor, the study adhered to the Consolidated Criteria for Reporting Qualitative Research (COREQ) checklist [12].

### Research team and reflexivity

The research team consisted of eight members: three physicians, one nurse qualitative-research expert, two economics/ business management specialists, and two public-health researchers. The principal investigator (SKA)—a male nurse with over ten years of clinical experience and training in qualitative inquiry—conducted most interviews. His insider knowledge of healthcare in the Kurdistan Region of Iraq (KRI) enriched contextual understanding but required ongoing reflexivity to limit bias. To support reflexive practice, the team held debriefing sessions after each interview to examine emerging themes, assumptions, and interpretations. A reflexive journal documented methodological decisions, emotional responses, and evolving insights. All team members contributed equally to coding and interpretation, ensuring cross-validation across disciplines. The interviewer had no supervisory relationship with participants, fostering neutrality and trust. The team’s gender and disciplinary diversity strengthened triangulation and credibility, ensuring findings reflected participants’ realities rather than researcher preconceptions. The research team also reflected on potential power dynamics related to professional roles, gender, and hierarchical positioning. As the primary interviewer was a nurse interviewing physician, this professional asymmetry may have influenced how participants framed their experiences or emphasized institutional challenges. To mitigate this, the interviewer adopted a non-evaluative stance, emphasized confidentiality, and encouraged open narrative accounts. Gender dynamics were also considered; although both male and female physicians participated, no systematic differences in disclosure were observed. These reflexive considerations were discussed during team debriefings and informed the interpretation of the data.

### Study setting and context

The study was conducted in the Kurdistan Region of Iraq (KRI), a semi-autonomous area that has faced recurrent fiscal crises since 2014 due to fluctuations in oil revenue, budget disputes with the federal government, and political instability. The region’s healthcare system comprises a dual structure of public and private facilities, characterized by limited resources, delayed salary payments, and growing dependence on out-of-pocket expenditure. The Kurdistan Region’s health financing context further illustrates the fragility of healthcare delivery during prolonged crisis. Health system funding follows a mixed public–private model, with high reliance on out-of-pocket payments and unstable public-sector financing, which have been repeatedly affected by salary delays and budget shortfalls [9, 13, 14]. Public-sector physicians are predominantly salaried, and recurrent fiscal disruptions have reduced payment reliability and institutional capacity, directly shaping workforce vulnerability and service provision [13, 14].

Data were collected between March and August 2024 across the governorates of Erbil, Sulaimaniyah, and Duhok. Sites included public tertiary hospitals, district-level hospitals, and private clinics, ensuring the inclusion of perspectives from both primary and specialized care. This period coincided with one of the most severe salary-delay episodes in the region, creating a natural setting to examine the impact of financial strain on healthcare delivery and workforce morale.

### Sampling and participants

Purposive maximum-variation sampling was employed to capture a broad range of perspectives by gender, specialty, years of experience, and employment sector. Inclusion criteria included: being a licensed physician currently practicing in KRI, having a minimum of five years of professional experience, and providing direct patient care during the economic crisis.

Twenty-five physicians (fifteen male, ten female), aged 30–56 years (mean 45.7), participated in the study. They represented various specialties, including internal medicine, pediatrics, surgery, obstetrics and gynecology, anesthesia, radiology, and psychiatry. Recruitment was conducted through hospital administrators, personal networks, and professional associations. Participants were fully briefed about the study’s purpose, confidentiality, and their right to withdraw at any time without penalty.

Data collection continued until thematic saturation was reached after the 23rd interview. Saturation was determined using the principle of informational redundancy, whereby no new codes or concepts emerged and existing codes recurred consistently across participants. Two additional interviews were conducted to verify saturation stability and confirm that no new themes were generated, ensuring credibility and completeness of the thematic structure [15, 16].

As the sample consisted exclusively of physicians, the findings primarily reflect medical practitioners’ perspectives on economic hardship, institutional responses, and professional resilience. Experiences of nurses, allied health professionals, and other frontline staff—who may face different forms of workload pressure, compensation structures, and organizational constraints—were not captured in this study. This focus may therefore shape the interpretation of resilience and healthcare delivery within a physician-centered professional lens.

### Data collection procedures

Data were gathered through a structured demographic questionnaire and in-depth semi-structured interviews designed to elicit detailed personal and professional narratives. The interview guide was developed following an extensive literature review and expert consultation to ensure comprehensive coverage of relevant domains. It was pilot-tested with two physicians to enhance clarity, sequencing, and cultural appropriateness.

Core areas of inquiry included workload and stress, salary instability and financial hardship, burnout and coping strategies, institutional and governmental responses, changes in clinical decision-making and patient management, and proposals for reform or innovation, including telemedicine and community-based initiatives (Table 1).

All interviews were conducted face-to-face in private hospital offices or clinics to preserve confidentiality and comfort. Each interview lasted between 40 and 70 min and was audio-recorded to ensure accurate data capture, with supplementary field notes documenting contextual observations and non-verbal cues.

Interviews were conducted in Kurdish and later transcribed verbatim by the lead researcher. Transcripts were professionally translated into English and then back-translated to ensure semantic equivalence. Discrepancies between translations were reconciled through consensus discussions involving bilingual experts, ensuring that meaning, tone, and contextual nuance were accurately retained.

**Table 1:** Interview guide questions

No	Interview guide questions
1	How has the economic crisis affected your clinical workload, availability of staff and resources, and your levels of stress or emotional strain?
2	How have institutional or governmental bodies responded to the crisis, and what forms of support (financial, staffing, training, or psychosocial) would most improve your ability to provide care?
3	Have resource limitations affected your clinical decision-making? If yes, please describe specific situations.
4	Have you observed any adaptive or innovative practices (e.g., telemedicine, community outreach, workflow changes) emerging during the crisis?
5	What reforms or system-level changes do you believe are necessary to strengthen healthcare delivery during prolonged economic instability?

### Ethical considerations

Ethical approval was granted by the Research Ethics Committee of the Faculty of Medicine, Koya University (Reference No. 12/37). All participants provided written informed consent after receiving both oral and written explanations of the study’s objectives, voluntary nature, confidentiality safeguards, and right to withdraw.

Participant anonymity was strictly maintained using alphanumeric identifiers (e.g., P1–P25), and all personal identifiers were removed from transcripts. Audio recordings, transcripts, and consent forms were securely stored on encrypted, password-protected devices accessible only to the research team. In accordance with institutional policy, all data will be permanently deleted five years after publication. No monetary incentives were offered to avoid coercion or bias.

### Data analysis process

Thematic analysis was performed following Ahmed’s et al. (2025) six-phase framework: data familiarization, initial coding, theme generation, theme review, theme definition, and report writing [11]. Coding focused on systematically organizing and refining data segments into coherent categories and themes through iterative comparison across interviews and clinical specialties [11]. Transcribed data were uploaded into NVivo 14 qualitative analysis software to facilitate systematic organization and retrieval. Two researchers (SKA and KAM) independently coded the first six transcripts to establish inter-coder reliability, which exceeded 85%. Inter-coder reliability was calculated using percent agreement generated through NVivo 14’s Coding Comparison Query, indicating a high level of consistency in code application across analysts. Any coding discrepancies were resolved through reflective dialogue until consensus was achieved.

Codes were developed inductively and progressively refined into themes through iterative comparison across interviews and clinical specialties [11]. A comprehensive codebook and thematic map were created to document the evolution of analytical decisions, ensuring a transparent audit trail. Member checking was conducted with five participants to validate interpretations and verify the authenticity of thematic representation. Illustrative quotations were selected to convey the depth and diversity of participants’ voices, emphasizing contextual meaning rather than frequency.

### Ensuring rigor, trustworthiness, and transparency

Methodological rigor was established through the application of Ahmed (2024) four trustworthiness criteria—credibility, transferability, dependability, and confirmability [17].

Credibility was achieved through triangulation of data sources (public and private sector physicians), member checking, and peer debriefing within the research team [17]. Transferability was strengthened by providing detailed contextual descriptions of the healthcare environment and participants’ professional settings [17]. Dependability was supported through the maintenance of a detailed methodological log, double recording, and version-controlled data management [17]. Confirmability was ensured through the use of reflexive note-taking and a transparent audit trail [17]. Authenticity and transparency were further enhanced through adherence to COREQ reporting standard [12]. The study was pre-registered in the Koya University Research Registry prior to data collection to promote accountability and traceability.

## Results

### Participant characteristics

**Fig. 1:**
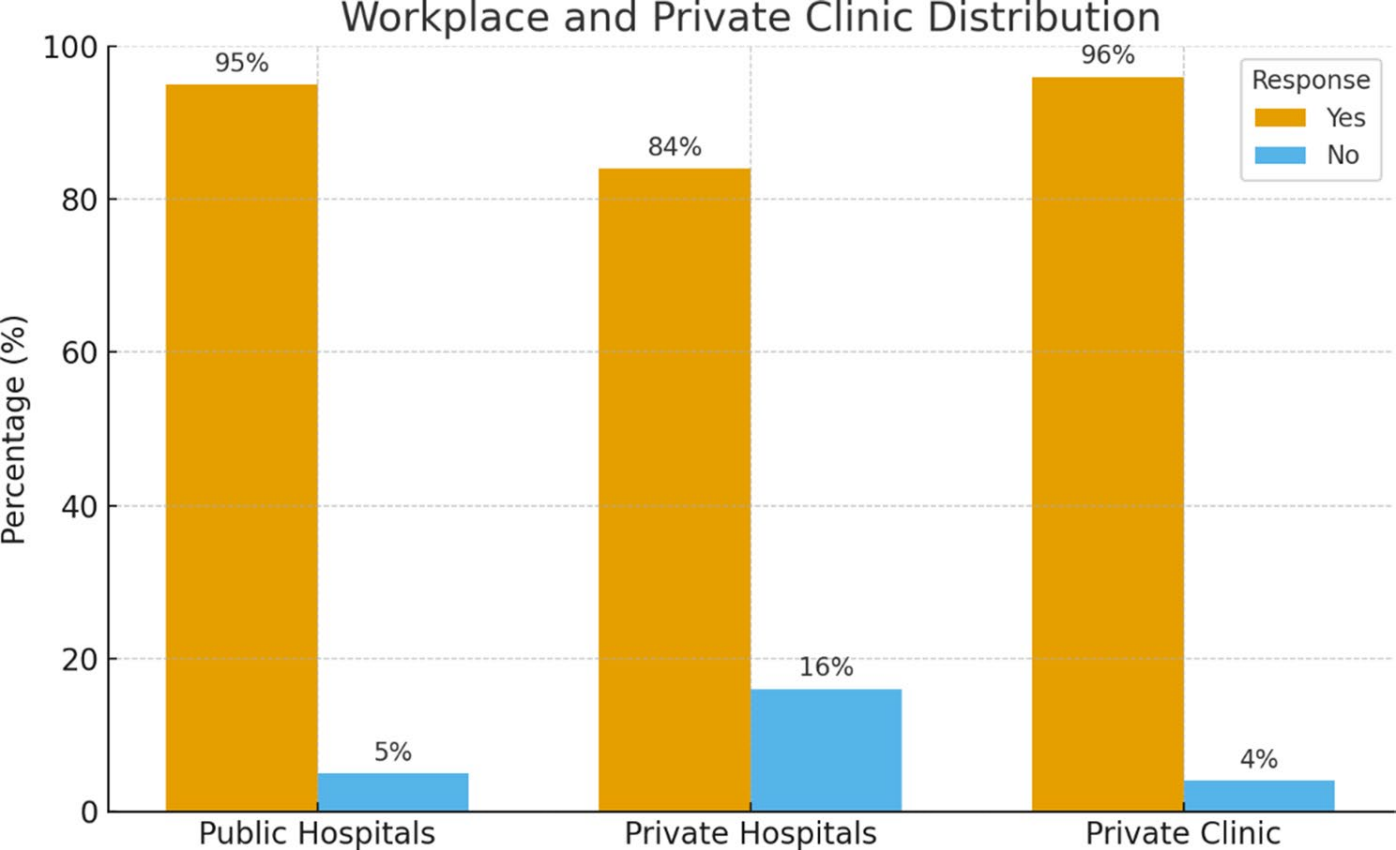
Workplace of the participants

Twenty-five physicians participated in the study, including 15 men (60%) and 10 women (40%), aged between 30 and 56 years (mean = 45.7 years), with an average of 20.6 years of professional experience. Participants represented diverse specialties—surgery (24%), obstetrics and gynecology (16%), internal medicine (8%), pediatrics (8%), and others. Most physicians (95%) were employed in the public sector, though a large proportion (84%) also worked in private hospitals, and nearly all (96%) operated private clinics to supplement income (Table 2; Fig. 1).

**Table 2:** Demographic and professional characteristics of participants (*n* = 25)

Variables	Category	*n* (%)
Gender	Male	15 (60)
	Female	10 (40)
Specialization	Surgery	6 (24.0)
	Obstetrics & Gynecology	4 (16.0)
	Medicine	2 (8.0)
	Urology	2 (8.0)
	Radiology	2 (8.0)
	Internal Medicine	2 (8.0)
	Rheumatologist	1 (4.0)
	Family Medicine	1 (4.0)
	General Surgery	1 (4.0)
	Cardiovascular and Thoracic Surgery	1 (4.0)
	Plastic Surgery	1 (4.0)
	Ophthalmology	1 (4.0)
	Pediatrics	1 (4.0)
Age in years (SD)	45.68 (8.73)	
Years of medical experience	20.64 (8.65)	
Years working in Kurdistan	19.24 (7.77)	

### Overview of thematic analysis

Thematic analysis generated ten interrelated but analytically distinct themes, capturing structural pressures on clinical work, psychological consequences for physicians, and the adaptive behaviors through which they sought to sustain care under prolonged economic strain. These include workload and stress, salary instability, psychological distress, coping mechanisms, institutional inaction, clinical decisions under constraint, proposed reforms, need for support, emerging innovations, and impact on patient care (Table 3).

**Table 3:** Summary of major themes, subthemes, and illustrative quotations

Themes	Subthemes	Illustrative quotes (Participant ID)
workload-related operational stress	Staff shortages in public sector; reduced private sector activity	“Staff are unpaid and overworked, leading to shorter hours and longer schedules.” (P7); “Less patients in private; sometimes I didn’t even go to my clinic.” (P11)
Salary instability	Delayed/reduced salaries; incentives cut	“Salaries went down and were delayed. This is the main problem.” (P4); “Yes, a lot since 2014, even with incentives cut.” (P2)
Psychological stress, burnout, and hopelessness	Demoralization, emotional exhaustion, discouragement	“Yes, a lot of my coworkers are unhappy and discouraged.” (P13); “I don’t know what to do anymore; it’s hopeless.” (P18)
Coping mechanisms	Volunteering; free services; semi-private models	“Most of the time, we try to help patients for free.” (P9); “We’re using a semi-private model to help doctors, nurses, and patients who don’t have many resources.” (P16)
Institutional inaction	Lack of system-level responses	“Nothing.” (P5); “No measures from institutions at all.” (P12)
Clinical decisions under constraints	Rationing care; postponing elective procedures	“Yes, we had to put off treatment because we didn’t have enough resources to handle difficult orthopedic cases.” (P21)
Proposed reforms	Salary stabilization; health insurance; systemic change	“Change the system.” (P6); “Health insurance and international support are necessary.” (P15)
Need for support	Financial, staffing, medical supplies, training	“We need a regular monthly budget and dependable paychecks.” (P10); “We need medical supplies and training programs for our staff.” (P19)
Emerging innovations	Telemedicine; community-based care	“There is some telemedicine expansion.” (P23); “Community-based care initiatives are being tried.” (P20)
Impact on patient care	Longer waiting times; reduced quality; negative outcomes	“There is a negative impact on health services, leading to a decline in quality of care.” (P8)

#### workload-related operational stress

This theme captures stress as an operational and structural phenomenon, arising from increased workload, staffing shortages, and service demands, rather than as a psychological outcome. This theme reflects how systemic workforce shortages translated into immediate and acute pressure on physicians’ daily functioning. The economic crisis has intensified physicians’ workloads in public hospitals while simultaneously reducing private sector activity, producing a dual strain on both productivity and income. Staff shortages forced longer shifts, fewer rest days, and task redistribution. These workload pressures frequently intersected with financial instability, creating a compounded strain that shaped physicians’ daily experiences across both public and private sectors.

*Staff are unpaid and overworked*,* leading to shorter hours and longer schedules.* (P7)

This account illustrates how structural underfunding simultaneously intensified workload in the public sector while eroding income opportunities in private practice, producing a dual pressure on both labor and livelihood.

#### Salary instability

Financial instability emerged not merely as an economic concern but as a structural force shaping physicians’ professional identity and stability. Salary delays, reductions, and unpredictable payment schedules were consistently identified as the most destabilizing aspect of the crisis. Participants described financial insecurity not as an isolated issue but as a compounding stressor that amplified the workload challenges reported in the previous theme. This instability undermined professional motivation, weakened institutional loyalty, and pushed many physicians to depend more heavily on private practice to sustain their livelihoods.

*Delayed and reduced salaries were the main problem.* (P4)

*Yes*,* a lot since 2014*,* even with incentives cut.* (P2)

#### Psychological stress, burnout, and hopelessness

In contrast to the previous theme, which addresses workload-related operational stress, this theme reflects the psychological consequences of prolonged exposure to structural pressures, including emotional exhaustion, burnout, and a pervasive sense of hopelessness. Participants’ narratives illustrated a progression from short-term stress responses toward deeper emotional exhaustion and burnout. Physicians described demoralization, emotional exhaustion, and a pervasive sense of hopelessness as defining features of the crisis period. Work pressure and financial hardship jointly contributed to burnout.

*I don’t know what to do anymore—it feels hopeless. *(P18)

This statement reflects not only emotional exhaustion but also a deeper erosion of professional meaning, where prolonged uncertainty transforms temporary stress into enduring burnout and moral fatigue.

#### Coping mechanisms

Whereas the previous themes describe sources of stress and its psychological consequences, this theme focuses on physicians’ active behavioral and ethical responses—how they adapted their practices to cope with institutional constraints and sustain care delivery. In the face of institutional stagnation, participants relied on self-organized coping strategies, including volunteering, free care for patients, and semi-private models designed to balance equity and sustainability.

*We often provide care for free to help patients.* (P9)

*We’re using a semi-private model to help doctors*,* nurses*,* and patients who don’t have many resources.* (P16)

These adaptive behaviors underscore a grassroots resilience, revealing how moral commitment and collective solidarity sustained basic healthcare delivery when systemic support was lacking.

#### Institutional inaction

Physicians consistently identified a gap between frontline needs and institutional responsiveness, underscoring weakened o institutional resilience. Nearly all participants criticized the absence of governmental or institutional responses to mitigate the effects of the economic crisis. Their statements—often brief and categorical—conveyed frustration and resignation.

*Nothing.* (P5)

*There were no institutional measures at all.* (P12)

This succinct account highlights physicians’ perception of systemic abandonment, where the absence of visible leadership action amplified feelings of isolation and eroded trust in organizational responsibility.

#### Clinical decisions under constraints

Resource shortages reshaped clinical reasoning, placing physicians in ethically complex decision-making environments. Limited staffing, financial barriers, and shortages of essential resources forced physicians to alter their clinical decision-making, frequently rationing care or postponing elective procedures.

*We postponed treatment due to insufficient resources for complex orthopedic cases.* (P21)

These constraints imposed ethical tension, as doctors navigated the dilemma between medical necessity and practical feasibility. The findings illustrate how economic austerity directly compromises clinical autonomy, eroding the foundation of patient-centered care.

#### Proposed reforms

Participants articulated forward-looking, system-level solutions that reflect a deep understanding of structural weaknesses. Participants proposed a range of reform strategies to restore system stability. The most urgent was salary stabilization, followed by the establishment of health insurance systems, international collaboration, and systemic restructuring.

*Change the system. *(P6)

*Health insurance and international support are necessary.* (P15)

These reform proposals reveal physicians’ awareness of the macro-structural dimensions of the crisis, recognizing that sustainability requires coordinated financial and institutional reform rather than short-term fixes.

#### Need for support

Calls for support highlighted the structural nature of the crisis and the need for both material and psychosocial reinforcement. Also, expressed a consistent call for financial, logistical, and professional reinforcement. Essential needs included regular salaries, medical supplies, training programs, and adequate staffing.

*We need a regular monthly budget and dependable paychecks.* (P10)

*We need medical supplies and training programs for our staff.* (P19)

This demand reflects a desire not merely for financial relief but for systemic reinforcement of capacity and competence—critical components of workforce retention and health system resilience.

#### Emerging innovations

Small-scale innovations represented informal resilience practices that helped sustain care in the absence of institutional action. Despite pervasive hardship, some physicians noted adaptive innovations, including the use of telemedicine and community-based outreach programs.

*There is some telemedicine expansion.* (P23)

*Community-based care initiatives are being tried.* (P20)

These developments, though nascent, indicate creative responses to structural collapse, suggesting an emerging culture of digital and community-driven care within Kurdistan’s healthcare system.

#### Impact on patient care

Participants emphasized that systemic disruptions ultimately translated into inequities in access and declines in care quality. All participants unanimously agreed that the economic crisis negatively affected patient care, resulting in longer waiting times, shortages of medication, and declining quality of service.

*These conditions reduced service quality and negatively affected patient care.* (P8)

This outcome represents the culmination of all preceding themes, illustrating how financial instability cascades into systemic inefficiency and compromised health outcomes.

Taken together, the ten themes illustrate a complex interplay between individual commitment and systemic vulnerability within the Kurdistan Region’s health system. Physicians consistently demonstrated strong *moral resilience*—sustaining professional values, adapting clinical practices, and supporting patients despite emotional strain and material shortages. In contrast, the near absence of coordinated institutional action revealed weak *institutional resilience*, with limited structural mechanisms to absorb shocks or support the workforce during crisis conditions. The convergence of themes shows how personal coping efforts, improvised innovations, and ethical decision-making compensated for systemic gaps, highlighting a health system that functions largely through the resilience of its frontline workers rather than through robust institutional scaffolding.

## Discussion

The findings demonstrate that the prolonged economic crisis in the Kurdistan Region of Iraq has reconfigured physicians’ professional lives through a convergence of financial insecurity, institutional fragility, and escalating emotional strain. Salary instability emerged as the central structural driver shaping both work patterns and morale. Physicians described delayed and reduced public-sector wages not as a temporary disruption but as a normalized condition since 2014, compelling widespread reliance on dual and multiple practice across public hospitals, private facilities, and personal clinics. This fragmentation of professional engagement intensified workload pressures while simultaneously exposing physicians to unstable patient flows and income uncertainty, weakening institutional attachment and accelerating demoralization. In this context, financial hardship was not merely an economic inconvenience but a catalyst for burnout, diminished professional meaning, and long-term workforce vulnerability. These dynamics mirror evidence from other crisis-affected systems in which austerity and fiscal instability undermine workforce stability and service continuity [13, 18, 19].

Interpreted through a resilience framework, the results reveal a pronounced asymmetry between individual and institutional capacity. Physicians consistently demonstrated moral and professional resilience—continuing to deliver care, improvising clinical solutions, and sustaining ethical commitment despite prolonged adversity. However, healthcare institutions exhibited limited institutional resilience. Participants repeatedly described an absence of coordinated leadership action, weak governance, and minimal structural support, in contrast to systems where organizational resilience, social support, and crisis-responsive policies buffer frontline staff and enhance performance [20–23]. As a result, the responsibility for system survival was effectively transferred to individuals, transforming resilience into a personal obligation rather than an institutional property. This imbalance magnified emotional exhaustion and reinforced a cycle in which workforce endurance compensated for systemic failure.

The psychological consequences of this structural neglect were evident in physicians’ accounts of burnout, hopelessness, and moral fatigue. Persistent workload pressures combined with financial insecurity produced not only emotional exhaustion but also a deeper erosion of professional purpose, consistent with conceptualizations of burnout as an occupational phenomenon driven by chronic organizational stressors [13]. Similar patterns have been reported in other crisis contexts, where prolonged economic strain and institutional instability intensify anxiety, depression, and disengagement among healthcare workers [10, 22–26]. Notably, participants described a paradox in which public-sector workloads increased while private-sector patient volumes declined, generating overextension in one setting and underutilization in another. This dual-sector strain heightens risks of attrition and disengagement, further weakening system capacity in already fragile conditions.

Despite these constraints, physicians enacted a range of adaptive strategies that reflect collective and value-driven coping. Volunteering, pro bono care, and the creation of semi-private models were frequently cited as mechanisms to sustain access for vulnerable patients and to preserve professional identity. These practices exemplify communal coping, whereby professionals pool efforts to confront shared adversity [27]. However, the findings also highlight the limits of such grassroots resilience. While individual and collective adaptation temporarily buffered service delivery, these efforts remain inherently fragile without institutional reinforcement. Consistent with evidence that resilience in healthcare systems must be structurally embedded rather than solely enacted by individuals [28], the Kurdistan case illustrates how moral commitment can sustain care in the short term but cannot substitute for durable organizational capacity.

Resource scarcity further reshaped clinical practice, compelling physicians to ration care, postpone elective procedures, and modify treatment decisions under financial and material constraints. These ethically distressing choices mirror experiences in other economically and politically fragile settings, where healthcare professionals are forced to balance clinical ideals against practical feasibility [29, 30]. Participants emphasized that such adaptations were not clinically driven but structurally imposed, with direct consequences for equity and quality of care. Evidence from Greece and other crisis-affected systems demonstrates that austerity and resource shortages deepen inequities, delay treatment, and disproportionately burden populations unable to access private alternatives [19, 31–34]. In the Kurdistan context, the normalization of rationing risks institutionalizing lower standards of care, eroding patient trust and reinforcing a bifurcated system in which access increasingly depends on financial means rather than clinical need.

Participants consistently identified salary stabilization as the most urgent prerequisite for workforce sustainability. Regular and predictable compensation is not only fundamental to livelihoods but also directly associated with morale, retention, and quality of care [35–37]. Beyond remuneration, physicians called for structural reforms, including the development of health insurance mechanisms to reduce out-of-pocket expenditure, strengthening of medical supply chains, and engagement with international partners to secure stable resources. These proposals align with evidence that well-designed financing reforms and resilient supply systems enhance equity, continuity, and crisis responsiveness, particularly in resource-constrained environments [38–40]. Importantly, such reforms would shift the burden of resilience from individuals to institutions, transforming resilience into a system-level capacity rather than an individual coping strategy.

Emerging innovations, including telemedicine and community-based care, were identified as nascent but promising responses to structural constraints. Although not yet widespread, these initiatives reflect adaptive potential within the system and echo global evidence that digital health and community-oriented models can expand access and mitigate inequities during periods of disruption [41, 42]. However, participants emphasized that innovation alone cannot compensate for persistent fiscal instability. Without foundational reforms in financing, governance, and workforce support, such initiatives risk remaining fragmented and unsustainable, rather than becoming integrated components of a resilient health system [43].

Collectively, this study advances understanding of health system resilience in fragile political and economic contexts by demonstrating that, in the Kurdistan Region, healthcare delivery is sustained primarily through physicians’ moral endurance rather than institutional capacity. While individual resilience and communal coping have preserved essential services, they simultaneously mask systemic vulnerability and risk normalizing precarity. The findings therefore extend existing literature by illustrating how prolonged economic crisis transforms resilience from an organizational attribute into a personal burden borne by frontline professionals. Sustainable healthcare delivery in such settings requires re-centering resilience at the institutional and policy level—through salary stabilization, transparent governance, workforce-centered support, and equitable financing—so that the capacity to absorb future shocks becomes a property of the system rather than a demand placed on individual physicians.

## Limitations and strengths

Some limitations of the research should be mentioned. The small sample size of twenty-five doctors means the findings are not generalizable to the healthcare system. Additionally, it is possible that participants’ expectations, or lack thereof, influenced their self-reported experiences.

Allied healthcare providers, pharmacists, and nurses, who may have contrasting experiences, have been largely disregarded in favor of physicians. Lastly, the use of a qualitative method poses challenges in tracking progress and establishing the relationship—if any—that exists between the economic crisis and its effects.

The study’s findings are clinically relevant and have far-reaching practical consequences, despite the study’s limitations. Consistency and predictability of pay is a determinant of employee satisfaction, retention, and the overall quality of service rendered. There is an urgent need for systemic improvements, including but not limited to, health insurance systems, medical supply chains, and international aid to strengthen resilience against future disasters. Burnout and mental health aid programs need to be prioritized to maintain the well-being and service provision of healthcare personnel. Access and equity can be improved through telemedicine and community healthcare initiatives. Given these effects, proactive measures can be taken by policymakers to enhance healthcare access equity in the immediate and distant future while fortifying resilience in response to economic volatility.

## Conclusion

This study illustrates the response of healthcare providers in the Kurdistan Region to the economic crisis, characterized by chronic underpayment, augmented workloads, heightened stress, and resource scarcity, while healthcare institutions have remained largely unresponsive. Regardless of such obstacles, healthcare workers demonstrate continued innovation and resilience. There is a pressing need for policymakers to invest strategically in stabilizing compensation, enhancing workforce wellness, and enacting systemic changes that serve to cultivate resilience. These efforts are critical, or else the dual practice model will likely exacerbate the inequities in healthcare access, while the erosion of care quality will persist unmitigated.

## Data Availability

All relevant data are within the paper and its supporting information files.

## Declarations

### Use of generative AI and AI-assisted technologies in the writing process

The authors declare that they used ChatGPT (OpenAI) to support the writing process of this article, specifically for language refinement, and improving clarity. The tool was employed to enhance the quality of expression, but did not contribute to the scientific content, data interpretation, or final conclusions of the manuscript. All AI-generated outputs were carefully reviewed, revised, and approved by the authors, who take full responsibility for the integrity and originality of the work. The use of AI adhered to ethical standards for scholarly publishing.
